# Shoulder Related Temperature Thresholds in FSSW of Aluminium Alloys

**DOI:** 10.3390/ma14164375

**Published:** 2021-08-05

**Authors:** David G. Andrade, Sree Sabari, Carlos Leitão, Dulce M. Rodrigues

**Affiliations:** 1 Institute for Sustainability and Innovation in Structural Engineering (ISISE), Department of Mechanical Engineering, University of Coimbra, 3030-788 Coimbra, Portugal; david.andrade@uc.pt (D.G.A.); dulce.rodrigues@dem.uc.pt (D.M.R.); 2 Centre for Mechanical Engineering, Materials and Processes (CEMMPRE), Department of Mechanical Engineering, University of Coimbra, 3030-788 Coimbra, Portugal; sree.sabari@dem.uc.pt; 3 Instituto Superior de Engenharia de Lisboa (ISEL), Department of Mechanical Engineering, Polytechnic Institute of Lisbon, 1959-007 Lisbon, Portugal

**Keywords:** aluminium alloys, temperature, FSSW

## Abstract

Friction Stir Spot Welding (FSSW) is assumed as an environment-friendly technique, suitable for the spot welding of several materials. Nevertheless, it is consensual that the temperature control during the process is not feasible, since the exact heat generation mechanisms are still unknown. In current work, the heat generation in FSSW of aluminium alloys, was assessed by producing bead-on-plate spot welds using pinless tools. Coated and uncoated tools, with varied diameters and rotational speeds, were tested. Heat treatable (AA2017, AA6082 and AA7075) and non-heat treatable (AA5083) aluminium alloys were welded to assess any possible influence of the base material properties on heat generation. A parametric analysis enabled to establish a relationship between the process parameters and the heat generation. It was found that for rotational speeds higher than 600 rpm, the main process parameter governing the heat generation is the tool diameter. For each tool diameter, a threshold in the welding temperature was identified, which is independent of the rotational speed and of the aluminium alloy being welded. It is demonstrated that, for aluminium alloys, the temperature in FSSW may be controlled using a suitable combination of rotational speed and tool dimensions. The temperature evolution with process parameters was modelled and the model predictions were found to fit satisfactorily the experimental results.

## 1. Introduction

Friction Stir Spot Welding (FSSW) is a solid-state joining technique, which was already proved to be suitable for the welding of aluminium alloys [1]. However, the understanding of the heat generation mechanisms and the prediction of the welding temperatures during the FSSW operation is still required for developing process parameters windows for a very diversified range of applications and base materials. Since FSSW is a solid-state joining technique, it is expectable that the welding thermal cycles may be fully controlled by a suitable selection of the tool characteristics and processing parameters.

**Figure 1:**
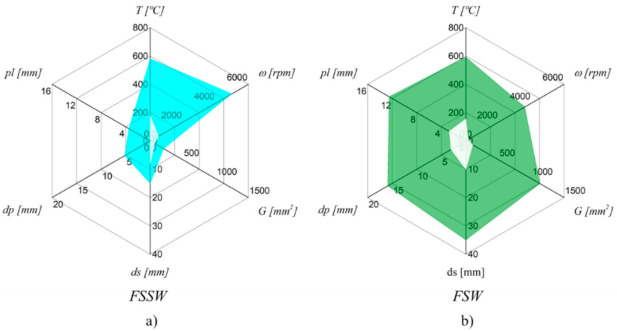
Range of welding temperatures (T) recorded and rotational speeds (ω), geometry parameters (G), pin lengths (p_l_), shoulder (d_s_) and pin (d_p_) diameters used in the works of Table 1, for the (**a**) FSSW and (**b**) FSW processes.

In Table 1 are listed works that studied the influence of the processing parameters on the thermal cycles in FSSW and Friction Stir Welding (FSW) of aluminium alloys. In Figure 1 is represented the range of temperatures (T) registered and of the rotational speeds (ω), pin lengths (p_l_) and shoulder (d_s_) and pin (d_p_) diameters used in the works listed in Table 1. The figure also shows the geometry parameter (G), which corresponds to the contact area between the tool and the workpiece, calculated for each of the tools tested as follows:

(1) $$G=\frac{\pi }{4}d_{p}^{2}+{\pi d}_{p}p_{l}+\frac{\pi }{4}\left(d_{s}^{2}-d_{p}^{2}\right)$$

Although, as stated by Rai et al., 2011 [2] and Janeczek et al., 2021 [3], the shape of the tool may have an influence on the welding outputs, such as welding temperature, material flow and joint strength, in order to simplify the calculation of G, for complex pin or shoulder geometries, G was calculated assuming an equivalent cylindrical geometry. According to Andrade et al. [4,5], G is a valuable parameter that takes into account the tool dimensions and can be related to welding outputs, such as the temperature and the torque. Analysing Figure 1, it is possible to conclude that a wider range of tool dimensions was tested in FSW, while a wider range of rotational speeds was tested in FSSW. Although the range of tool dimensions and rotational speeds tested in FSW and FSSW was different, the temperatures registered in the different works varied in the same range, i.e., between 200 and 600 °C.

**Figure 2:**
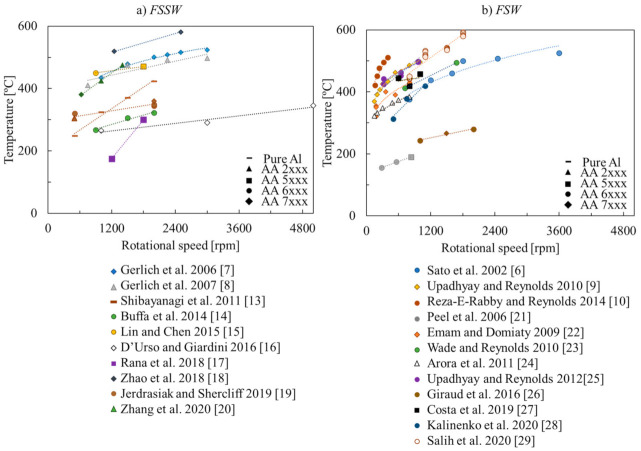
Evolution of the temperature with the rotational speed for the experimental works considered in Table 1 on (**a**) FSSW and (**b**) FSW [6,7,8,9,10,13,14,15,16,17,18,19,20,21,22,23,24,25,26,27,28,29].

**Figure 3:**
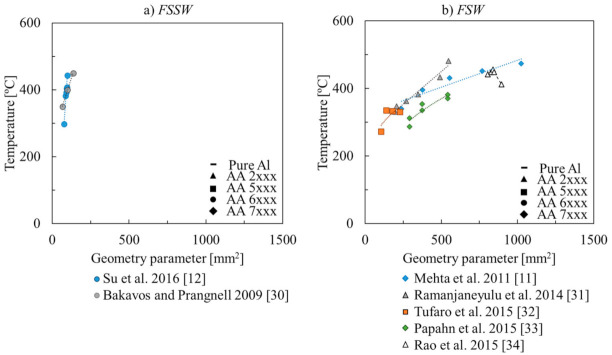
Evolution of the temperature with the geometry parameter for the experimental works considered in Table 1 on (**a**) FSSW and (**b**) FSW [11,12,30,31,32,33,34].

Figure 2 and Figure 3 show in more detail the welding temperatures registered in each work in Table 1, as a function of the rotational speed and tool dimensions, respectively. For most of the works in the figures, it is possible to observe that the welding temperature increased with the rotational speed. Although, some authors [6,7,8,9,10], reported that the welding temperature increases non-linearly with the rotational speed, stabilising for temperatures close to the melting temperature of the alloy being welded. This was attributed by Gerlich et al. [7,8] to the occurrence of incipient local melting, which induces slipping contact conditions at the tool–workpiece interface, reducing the heat generation. Upadhyay and Reynolds 2010 [9] also stated that, with the increase of the welding temperature, there is a decrease in the base material flow stress, which limits the power generation by plastic dissipation. On the other hand, as shown in Figure 3, the welding temperature also increases with the tool dimensions. According to Mehta et al., 2011 [11] and Su et al., 2016 [12], the temperature increases with the tool dimensions due to the increase in frictional and mechanical work.

Although in all the works listed in Table 1, it was depicted an evolution of the welding temperature with the rotational speed and/or the tool dimensions, no global trend in the temperature evolution with those process parameters may be observed when analysing Figure 2 and Figure 3. This may be related to the fact that neither the temperature measurement techniques nor the temperature measurement positions were similar in the different works, which may have had influence on the maximum temperatures registered by the different authors. The heat dissipation conditions may also had been very different among the different works. Finally, no specific correlation between the welding temperatures registered and the aluminium alloy being welded can also be inferred from the analysis of the figures.

**Table 1:** Experimental works that analyse the influence of the processing parameters on the welding thermal cycles registered during the FSSW and FSW processes of aluminium alloys.

FSSW		FSW	
Author	Base Material	Author	Base Material
Gerlich et al., 2006 [7]	AA7075	Sato et al., 2002 [6]	AA6063
Gerlich et al., 2007 [8]	AA2024	Peel et al., 2006 [21]	AA5083, AA6082
Bakavos and Prangnell 2009 [30]	AA6111	Emam and Domiaty 2009 [22]	AA7050
Shibayanagi et al., 2011 [13]	Pure Al	Upadhyay and Reynolds 2010 [9]	7050
Buffa et al., 2014 [14]	AA6082	Wade and Reynolds 2010 [23]	AA6019
Lin and Chen 2015 [15]	AA5052, AA6061	Mehta et al., 2011 [11]	AA7075
D’Urso and Giardini 2016 [16]	AA7050	Arora et al., 2011 [24]	AA2524
Su et al., 2016 [12]	AA6061	Upadhyay Reynolds 2012 [25]	AA6056
Rana et al., 2018 [17]	AA5052	Ramanjaneyulu et al., 2014 [31]	AA2014
Zhao et al., 2018 [18]	AA7B04	Reza-E-Rabby and Reynolds 2014 [10]	AA 6061
Jedrasiak and Shercliff 2019 [19]	AA2024-T3, AA6082-T6, AA7449-T3	Tufaro et al., 2015 [32]	AA5052
Zhang et al., 2020 [20]	AA2024, AA7075	Papahn et al., 2015 [33]	AA7075
-	-	Rao et al., 2015 [34]	AA2019
-	-	Giraud et al., 2016 [26]	AA7020, AA6060
-	-	Costa et al., 2019 [27]	AA5754
-	-	Kalinenko et al., 2020 [28]	AA6061
-	-	Salih et al., 2020 [29]	AA6082

In the current work, a parametric analysis of the influence of the tool diameter, rotational speed and base material characteristics on the welding temperatures, in FSSW, was conducted. The plunging and dwelling phases of this process were simulated by performing bead on plate spot welds in thick AA2017, AA5083, AA6082 and AA7075 aluminium alloy plates. Due to the prominent role of the tool shoulder on the heat generation [35,36,37], only pinless tools, with different diameters, were used in the investigation. By performing bead on plate spot welds in thick plates, it was possible to fully capture the size and morphology of all the process affected zones.

## 2. Experimental Procedure

**Figure 4:**
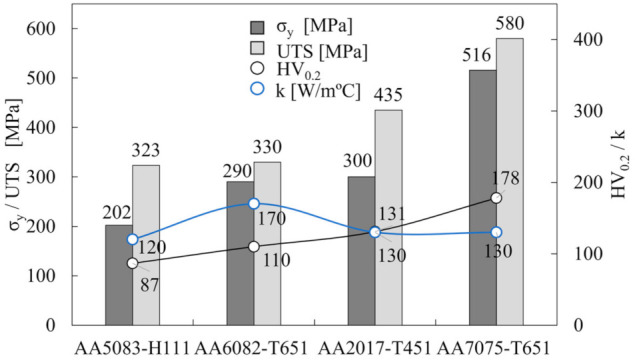
Base material properties: yield strength (σ_y_), ultimate tensile strength (UTS), hardness (HV_0.2_) and thermal conductivity (k).

In this work, the temperature evolution during the plunging and dwelling phases of the FSSW process was analysed by producing bead-on-plate spot welds, in 100 × 100 × 10 mm aluminium alloy rolled plates. Three heat treatable (AA2017-T451, AA6082-T651 and AA7075-T651) and one non-heat treatable (AA5083-H111) aluminium alloys were used in the investigation. The chemical composition of the alloys is presented in Table 2. In Figure 4 are compared the yield strength (σ_y_), ultimate tensile strength (UTS), hardness (HV_0.2_) and thermal conductivity (k) for each aluminium alloy. As it is possible to conclude from the figure, the four alloys tested had very different strength but similar physical properties.

The welds were produced in a MTS I-STIR PDS [38] machine, in position control, using pinless tools with flat shoulders, in a three-stage welding operation. First, the plunging phase, in which the tool was moved vertically with a plunging speed of 0.125 mm/s, until a 0.5 mm depth was reached. The next stage consisted of the dwelling phase, during which the rotating tool remained in contact with the workpiece during 60 s. Finally, the third stage consisted of the tool removal. The 60 s dwelling time was used to ensure that steady-state conditions, in heat generation, were reached. The welding parameters, which are summarised in Table 3, were chosen in order to investigate the influence of varying rotational speeds, between 660 to 1500 rpm, shoulder diameters, between 10 to 18 mm, and base material properties, on the heat generation during welding.

The influence of the tool material on the heat generation was also analysed by testing uncoated and coated WC tools. Monolithic CrAlN and CrAlAgN coatings and a multi-layered CrAlN/TiAlN film were tested for the coated tools. The CrAlN base coatings were deposited by reactive magnetron sputtering, working in unbalanced mode, onto WC tools. The chemical composition, mechanical properties and thickness of the coatings are displayed in Table 4. The coatings were produced in 12 mm diameter tools which were tested in the welding of the AA6082 aluminium alloy at 660 rpm. The selection of the base material for testing the coated tools was based on previous works from the authors Leitão et al., 2012 [39] and Sabari et al., 2020 [40], which showed that this alloy experience intense flow softening during welding, a characteristic favourable for ensuring that the coating was not destroyed/removed during welding. The tool diameter was also selected based on previous studies on the heat generation in FSSW of steels, performed by Andrade et al. [37,41], which indicated that no saturation in heat generation would occur when welding with this tool diameter.

A FLIR A655sc thermographic camera was used to record the welding temperatures, and the thermal cycles were analysed following the practices proposed in [37]. The temperatures were measured in the tool shank, which is exactly above the contact with the base material. The tools’ thermal emissivity was calculated by heating the tools in a furnace, up to temperatures in the same range of the ones experienced during welding. The heating temperatures were monitored using thermocouples and compared with the temperatures obtained with the thermographic camera by adjusting the thermal emissivity until it reaches the values shown by thermocouples. An average emissivity of 0.7 was determined and used in all welding trials. During the welding process, the instantaneous evolution of the spindle torque was also measured, in order to assess possible differences in the mechanical response of the base materials to the different welding conditions.

**Figure 5:**
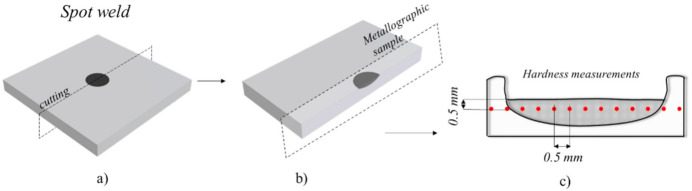
Schematic representation of the samples preparation after welding: (**a**) cutting location, (**b**) metallographic sample and (**c**) hardness measurements scheme.

As schematically represented in Figure 5, after welding, the specimens were stored at room temperature and metallographic samples were extracted from the welds cross section, using a cutting bandsaw machine (Figure 5a), and polished according to standard procedures (Figure 5b). Vickers micro-hardness measurements were carried out at 0.5 mm from the weld top surface with 0.5 mm spacing between indentations, using 200 g load and 15 s holding time (Figure 5c).

## 3. Results and Discussion

### 3.1. Temperature Evolution during the Welding Process

**Figure 6:**
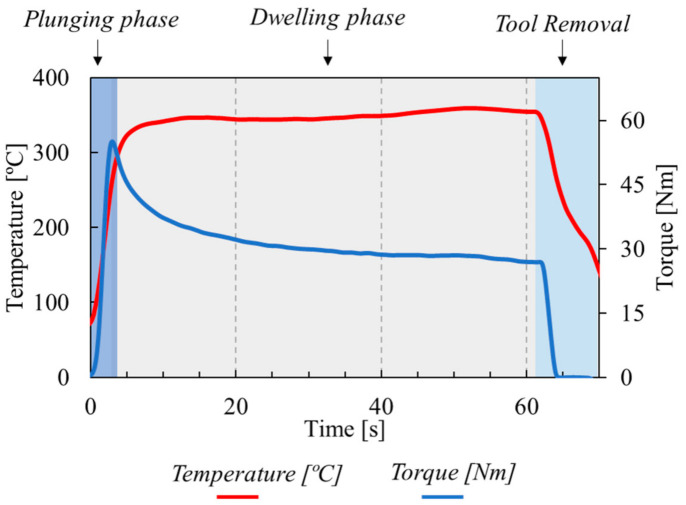
Evolution of temperature and torque with time for the weld produced in the AA7075 aluminium alloy, with a tool diameter of 16 mm and a rotational speed of 660 rpm.

The evolution of the temperature and torque with time, registered for a weld produced in the AA7075 aluminium alloy, with a tool diameter of 16 mm and a rotational speed of 660 rpm, is shown in Figure 6. Analysing the figure, it is possible to conclude that, at the beginning of the welding process, the temperature rapidly increased, at a rate of 50 °C/s, as the tool was plunged into the workpiece. The steep increase of the temperature stopped just after the plunging phase was finished, and after that, the temperature remained in an almost constant value, until the end of the process. Since the temperature variation during most of the dwelling period was lower than 4 °C/s, the FSSW temperature was calculated as the average of the temperatures registered during the dwelling period.

As for the temperature, the torque also increased at the beginning of the welding process, reaching a maximum value when the tool was plunged to the prescribed value. However, contrary to that registered for the temperature evolution, at the end of the plunging phase, the torque started to decrease, continuing to decrease during part of the dwelling phase. The decrease in torque shows that the mechanical interaction between the tool and the workpiece, and in this way, the heat generation mechanisms, evolve during the dwelling stage of the welding process. Actually, a decrease in the heat generation during the dwelling period is the only explanation for the very small change in temperature during this stage of the welding process. After the tool removal, the temperature and torque instantaneously started to decrease. The temperature and torque evolution illustrated in Figure 6 was identical for all the welds produced, independently of the welding parameters and/or of the aluminium alloy tested.

### 3.2. Temperature Evolution with Process Parameters

**Figure 7:**
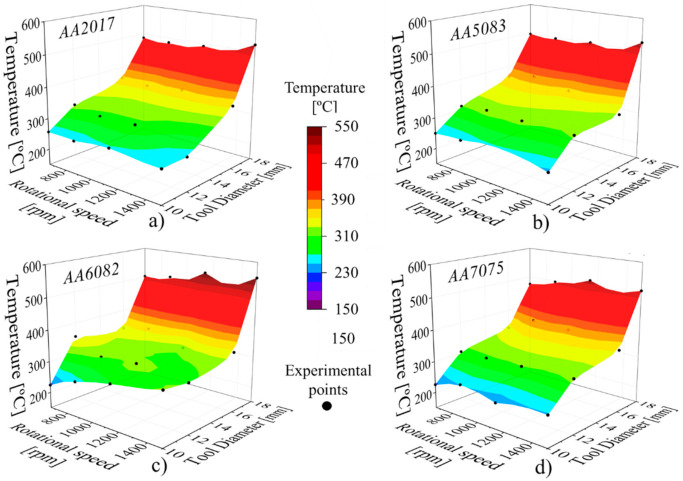
Evolution of the temperature values with the rotational speed and tool diameter for the (**a**) AA2017, (**b**) AA5083, (**c**) AA6082 and (**d**) AA7075 aluminium alloys.

The evolution of the FSSW temperature values, determined from the thermal cycles, with the rotational speed and the tool diameter, for all the aluminium alloys tested are plotted in Figure 7. The 3D surfaces were plotted using the commercial software package Origin by interpolation of the experimental data. Analysing the figure, it is possible to conclude that, for each base material, the FSSW temperature increases non-linearly with the tool diameter, irrespective of the rotational speed used. Increasing the tool diameter from 10 to 18 mm resulted in an increase of around 250 °C in the welding temperature, for all the aluminium alloys tested. On the other hand, the figure also shows that independent of the shoulder diameter or aluminium alloy tested, the temperature almost did not vary with the rotational speed, showing that, for aluminium alloys, 600 rpm is the limit rotational speed for which the heat generation stabilises. However, the figure also shows that the temperature stabilisation with the rotational speed does not only occurs when the melting temperature of the alloys is approached, as stated in some literature. Actually, the results show that there is a temperature threshold associated with each tool diameter and that this temperature threshold is independent of the rotational speed, at least for rotational speeds higher than 600 rpm.

In a previous investigation from the current authors [37,41] on the FSSW of steels, it was also demonstrated that the FSSW temperatures increase with the tool diameter, which was found to be the main factor governing the heat generation. According to those works, for steels, the welding temperatures only increase with the rotational speed for tool diameters smaller than 12 mm, while for tool diameters larger than 16 mm, no variation of the welding temperature was registered for rotational speeds higher than 870 rpm. Comparing current results, with that obtained for steels, it is possible to conclude that the welding conditions for which a shoulder related temperature threshold is registered are different for ferrous and aluminium alloys, but are similar among the different aluminium alloys.

**Figure 8:**
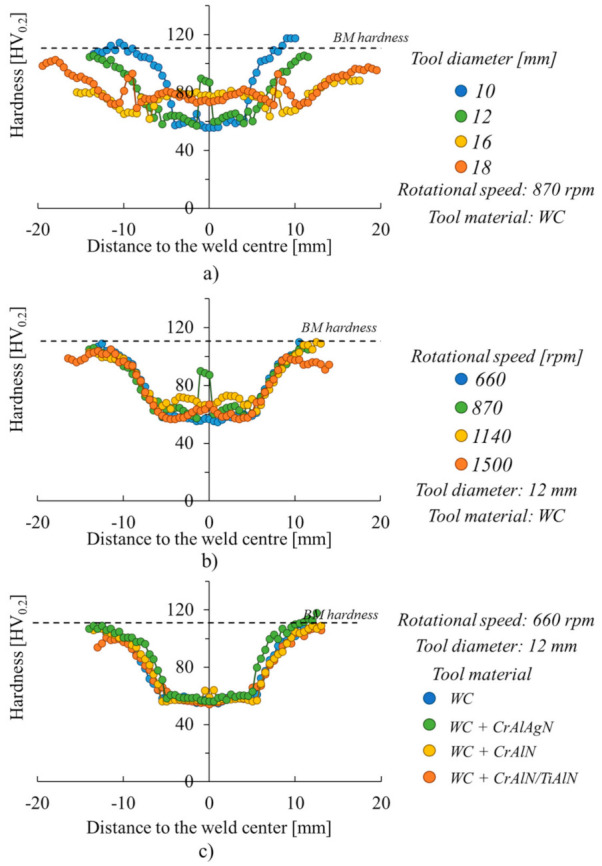
Hardness profiles for the welds produced with the AA6082 aluminium alloy with (**a**) the uncoated tool, at constant rotational speed of 870 rpm and varied tool diameters between 10 to 18 mm, (**b**) with the uncoated tool, at constant tool diameter of 12 mm, and varied rotational speed between 660 to 1500 rpm and (**c**) with the WC uncoated tool and the WC tools coated with the CrAlN, CrAlAgN and CrAlN/TiAlN films, and a constant tool diameter and rotational speed of 12 mm and 660 rpm, respectively.

In order to check if the reported trends on the FSSW temperature evolution, with the tool diameter and rotational speed, were not related with inaccuracies in the temperature acquisition during welding, hardness measurements were performed in the cross section of the welds produced in the heat treatable AA6061-T651 aluminium alloy, which according to MacKenzie et al., 2016 [42], was the one with the lowest quenching sensitivity among all the alloys tested. Figure 8 compares the hardness profiles for the welds produced with varied tool diameters, rotational speeds and tool coatings. More precisely, shown in Figure 8a are the hardness profiles for the welds produced with a constant rotational speed of 870 rpm, and varied tool diameters between 10 to 18 mm. Analysing the figure, it is possible to conclude that the hardness values varied according to the tool diameter, which proves that the base material was subjected to different thermal cycles when welding with the different tools.

Shown in Figure 8b is the evolution of the hardness profiles with the rotational speed. The hardness profiles displayed in the figure correspond to the welds produced with the 12 mm diameter tool and rotational speeds between 660 to 1500 rpm. Analysing the figure, it is possible to conclude that the evolution of the hardness with the distance to the weld centre is very similar to that shown in Figure 8a. However, in Figure 8b, no important differences in the hardness values may be observed for the welds produced with different rotational speeds. This result shows the small influence of the rotational speed on the welding thermal cycles.

In Figure 8c are compared the hardness profiles for the welds produced with the uncoated WC tool and with the CrAlN, CrAlAgN and CrAlN/TiAlN coated tools, with a constant rotational speed and tool diameter of 660 rpm and 12 mm, respectively, in the AA6082 aluminium alloy. The figure shows that all the hardness profiles are similar, indicating that the heat generation was similar for all the tools, irrespective of its characteristics. This result enables to conclude that the shoulder diameter dependent temperature threshold is independent of the tool material and only varies with the tool dimensions.

The previous results show that, independent of the aluminium alloy being welded, for rotational speeds higher than 600 rpm, the welding temperatures may be previewed/controlled by an appropriate choice of the tool diameter. To the current authors’ knowledge, no previous work has reported the same type of conclusions. However, as shown in Figure 2, some other authors [15,16,19,27] also reported a very small variation of the welding temperature, with the increase of the rotational speed, for rotational speeds higher than 600 rpm.

### 3.3. Torque Evolution with Process Parameters

**Figure 9:**
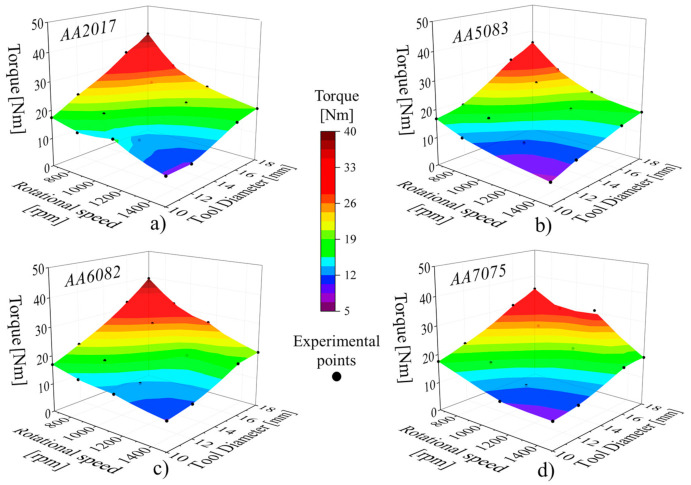
Evolution of torque with the rotational speed and tool diameter for the (**a**) AA2017, (**b**) AA5083, (**c**) AA6082 and (**d**) AA7075 aluminium alloys.

To better understand the influence of the process parameters on the heat generation, the thermomechanical conditions developed during welding were assessed by analysing the tool torque. In Figure 9 is now plotted the evolution of the torque values, determined from the torque curves, with the rotational speed and the tool diameter. In order to be able to correlate the torque with the temperature, the average torque values were calculated considering the same time interval used to determine the average temperatures. Analysing the figure, it is possible to conclude that irrespective of the aluminium alloy being welded, for constant tool rotational speeds, the torque increases with the tool diameter, and for constant tool diameters, the torque decreases by increasing the rotational speed. It is also possible to observe that the influence of the rotational speed on torque is stronger for larger than for smaller tool diameters. On the other hand, the influence of the tool diameter on torque is stronger for lower than for higher rotational speeds. Considering that, for each tool diameter, the temperature does not change significantly with the rotational speed, as shown in Figure 7, but the torque significantly decreases, it is possible to conclude that, for each tool diameter, the mechanical interaction between the tool and the workpiece, and in this way, the heat generation mechanism, are different for the different rotational speeds.

**Figure 10:**
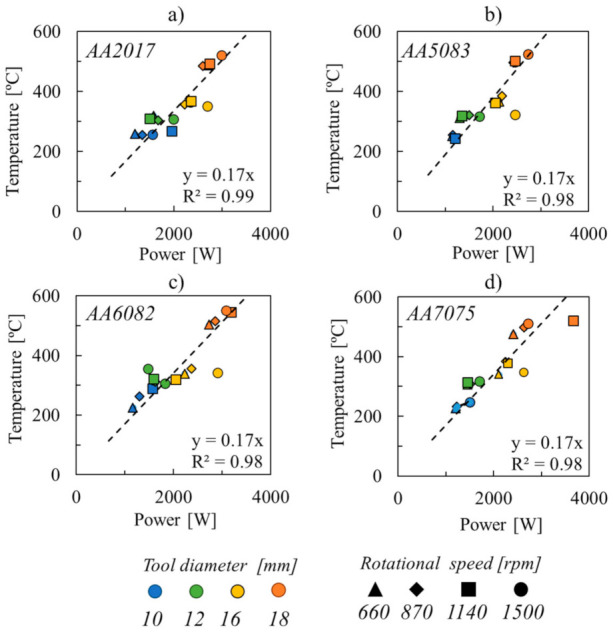
Evolution of temperature with the welding power, for the (**a**) AA2017, (**b**) AA5083, (**c**) AA6082 and (**d**) AA7075 aluminium alloys.

Shown in Figure 10 is the evolution of the temperature with the welding power, calculated by multiplying the tool torque (M) by the angular velocity

(2) $$P=\frac{2\pi \times \omega }{60}\times M,\;$$

for all the alloys and welding conditions tested. Analysing the figure, it is possible to observe a linear relationship between temperature and power. Moreover, as shown by the trend lines plotted in the graph of the figure, the slope between temperature and the welding power is equal for all the aluminium alloys. These results also support the previous assumption that the thermomechanical conditions developed during welding may evolve with process parameters.

### 3.4. Modelling Temperature

A relationship between the process parameters and the welding temperatures was already established and validated by Andrade et al., 2020 [4] for the FSW of aluminium alloys:

(3) $$\left\{\begin{array}{c} T=\lambda \;\times K_{T}C_{T}^{\varphi }\;\mathrm{for}\;C_{T}<20000\\ T=\lambda \times 590{}^{\circ}C\;\mathrm{for}\;C_{T}\geq 20000 \end{array}\right.$$

In the previous equation, λ is a constant that considers the influence of the different experimental apparatus in acquiring the maximum temperature, such as differences in temperature measurement techniques, the position at which the temperature was measured relative to the weld axis, backing plate material, among others. K_T_ and φ are constants which were determined to be equal to 50 and 0.25, respectively. C_T_ is the temperature coefficient given by

(4) $$C_{T}=\frac{G\omega }{\sqrt{\mathrm{vt}}}\;$$

where ω is the rotational speed, v is the traverse speed, t is the plate thicknesses and G is the geometry parameter (Equation (1)). In accordance with current paper results, on the evolution of the FSSW temperature with the processing parameters, the rotational speed is a secondary parameter governing the thermal cycles. So, for rotational speeds higher than 600 rpm, the previous temperature model is not suitable for predicting the welding temperatures. In this way, the temperature coefficient $C_{T}\;$ was adapted by withdrawing the traverse speed from its formulation and by considering the important influence of the rotational speed on the heat generation threshold. Thus, a new coefficient, C_T,SW_, is proposed.

(5) $$\left\{\begin{array}{c} C_{T,\mathrm{SW}}=\frac{G\omega }{\sqrt{t}},\;\mathrm{for}\;\omega <\omega_{\mathrm{crit}}\\ C_{T,\mathrm{SW}}=\frac{{G\omega }_{\mathrm{crit}}}{\sqrt{t}},\;\mathrm{for}\;\omega \geq \omega_{\mathrm{crit}}, \end{array}\right.\;$$

where ω_crit_ is the critical rotational speed for which no variation in temperature occurs. According to current results, ω_crit_ is equal to 600 rpm for aluminium alloys.

**Figure 11:**
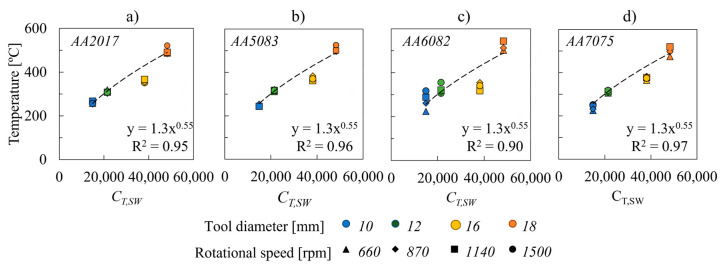
Evolution of temperature with the C_T,SW_ coefficient for the (**a**) AA2017, (**b**) AA5083, (**c**) AA6082 and (**d**) AA7075 aluminium alloys.

Plotted in Figure 11 are the welding temperatures versus the temperature coefficient (C_T,SW_*)*. The figure clearly shows that C_T,SW_ coefficient reproduces the evolution of the welding temperature satisfactorily through the relationship

(6) $$T=K_{T,\mathrm{SW}}\times C_{T,\mathrm{SW}}{}^{\varphi {,}_{\mathrm{SW}}},$$

in which K_T,SW_ and φ,_SW_ are constants. Fitting the experimental results, it was determined that K_T,SW_ and φ,_SW_ are equal to 1.3 and 0.55, respectively, for all the aluminium alloys.

The accurate prediction of the experimental results, for the very large range of welding conditions and base materials tested, proves that the C_T,SW_ coefficient is reliable for predicting the evolution of the temperature, with the process parameters, in spot welding of aluminium alloys. Previewing the welding temperature from process parameters is very important since it enables the fine tuning of the process parameters for producing welds at a desired temperature, without the need of performing expensive and time-consuming trial and error tests.

## 4. Conclusions

In the present work, the influence of the tool diameter, rotational speed, base material and tool characteristics, on the heat generation in FSSW, was analysed. The following conclusions were reached:

- For rotational speeds higher than 600 rpm, the welding thermal cycles may be fully controlled by an appropriate selection of the tool diameter. For each tool diameter, a threshold in the welding temperatures is reached, independent of the rotational speed, tool material and/or aluminium alloy being welded.
- The shoulder related temperature threshold is attained for temperatures far below the melting temperature of the alloy being welded and increase with the increase of the tool diameter. Increasing the tool diameter from 10 to 18 mm resulted in an increase of the welding temperature of around 250 °C. The temperature evolution with the process parameters was found to be similar among the different aluminium alloys.
- The tool torque was found to decrease with the increase of the rotational speed and with the decrease of the tool diameter. For the 10 and 18 mm tool diameters, increasing the rotational speed from 660 to 1500 rpm, resulted in a decrease in torque of around 7 and 20 Nm, respectively. On the other hand, a linear relation was observed between the welding power and the temperature for all the aluminium alloys (M=0.17×P).
- An analytical coefficient, C_T,SW_, was determined for calculating the temperature from process parameters, which provide accurate temperature predictions for the very large range of welding conditions and base materials tested. The model constants were found to be similar among the different aluminium alloys ($K_{T,\mathrm{SW}}=1.3\;\mathrm{and}\;\varphi {,}_{\mathrm{SW}}=0.55)$.

## Figures and Tables

**Table 2:** Chemical composition of the aluminium alloys (weight %).

Chemical Composition (Weight %)	Si	Fe	Cu	Mn	Mg	Cr	Zn	Ti	Al
AA2017-T451	0.2–0.8	0.7	3.5–4.5	0.4–1	0.4–1	0.1	0.25	0.25	Rem.
AA5083-H111	0.4	0.4	0.1	0.4–1	4–4.9	0.05–0.25	0.25	0.15	Rem.
AA6082-T651	0.7–1.3	0.5	0.1	0.4–1	0.6–1.2	0.25	0.2	0.1	Rem.
AA7075-T651	0.4	0.5	1.2–2	0.3	2.1–2.9	0.18–0.28	5.1–6.1	0.2	Rem.

**Table 3:** Welding parameters.

Base Material	Tool Diameter [mm]	Tool Material	Rotational Speed [rpm]	Dwell Time [s]
* 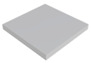 *	* 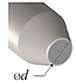 *	* 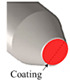 *	* 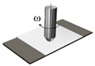 *	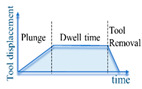
AA2017-T451 AA5083-H111 AA6082-T651 AA7075-T651	10, 12,16, 18	WC	660, 870, 1140, 1500	60
AA6082-T651	12	WC+ CrAlN WC+ CrAlAgN WC+ CrAlN/TiAlN	660	60

**Table 4:** Chemical composition, mechanical properties and thickness of the coatings.

Coating	CrAlN	CrAlAgN	CrAlN/TiAlN
Chemical Composition (at.%)	Cr—36.2 Al—14.2 N—49.6	Cr—30.3 Al—9.8 Ag—10.3 N—49.7	Cr—24.3 Al—14.8 Ti—13.3 N—47.5
Hardness (GPa)	18	20	20
Young’s Modulos (GPa)	280	251	469
Elastic strain to failure (H/E) parameter	0.064	0.080	0.043
Coating thickness (µm)	2.6	3.1	3.5

## Data Availability

The data underlying this article will be shared on reasonable request from the corresponding author.
